# Enhanced hybrid pre-coding and power allocation algorithms for smart irrigation systems using OFDM-based WSNs

**DOI:** 10.1371/journal.pone.0321283

**Published:** 2025-05-13

**Authors:** Emad S. Hassan

**Affiliations:** Department of Electrical and Electronics Engineering, College of Engineering and Computer Science, Jazan University, Jizan, Saudi Arabia; Thamar University: Dhamar University, YEMEN

## Abstract

Power allocation combined with pre-coding techniques is still an emerging field, with many challenges yet to be resolved. This paper contributes to filling this gap by proposing and evaluating hybrid algorithms that integrate pre-coding with low-complexity power allocation techniques for Orthogonal Frequency Division Multiplexing (OFDM)-based Wireless Sensor Networks (WSN) in smart irrigation systems. The use of linear pre-coding provides an efficient and simple solution to mitigate channel fading. By exploiting the channel’s frequency selectivity, the power allocation algorithms adjust the modulation type and power distribution for each sub-carrier dynamically. As a result, the proposed hybrid algorithms surpass static schemes, offering notable improvements in system performance. These algorithms adjust both the signal constellation size and power distribution based on the Signal-to-Noise Ratio (SNR) values observed across the sub-carriers. Additionally, practical considerations like Rate Maximization (RM) are incorporated to provide flexibility for various application needs. Extensive simulations validate the effectiveness of the proposed algorithms in minimizing power consumption and boosting performance in OFDM-based WSNs for smart irrigation. Numerically, the proposed algorithms can reduce the required SNR by up to 18 dB for a target throughput of 400 bits/symbol and outperforming conventional algorithms in terms of throughput, energy efficiency, and network lifetime, with the pre-coded Greedy power allocation (pre-GPA) algorithm delivering up to 98.5% throughput and the longest system lifespan.

## 1. Introduction

Smart irrigation systems have emerged as a promising solution for efficient water management in agricultural practices [1]. By leveraging wireless sensor networks (WSNs), these systems enable real-time monitoring of soil moisture levels, weather conditions, and plant health, allowing for precise and targeted irrigation. However, the success of smart irrigation systems critically depends on the energy efficiency and longevity of the deployed WSNs [2–8]. In WSN-based smart irrigation systems, sensor nodes are responsible for collecting and transmitting environmental data, which requires a continuous supply of power. The limited energy resources available to these nodes pose significant challenges in ensuring long-term system operation. Therefore, achieving energy efficiency and prolonging the sensor nodes’ lifetime are fundamental objectives for enhancing the performance of smart irrigation systems [1,2]. To address these challenges, researchers have focused on developing energy-efficient algorithms and techniques for power allocation in WSNs. By dynamically adapting the power allocation based on the environmental conditions and communication requirements, these algorithms aim to optimize energy consumption while maintaining system performance.

Orthogonal Frequency Division Multiplexing (OFDM) has gained considerable attention as a modulation scheme for WSNs due to its robustness against multipath fading and interference [9–11]. OFDM-based WSNs offer a powerful solution for improving the performance and energy efficiency of WSNs [12]. OFDM is a modulation scheme that divides the available frequency band into multiple sub-carriers, each carrying a portion of the data. By leveraging the frequency selectivity of the channel, OFDM enables robust communication by mitigating the effects of multipath fading and interference [13]. In the context of WSNs, the adoption of OFDM brings several advantages. Firstly, OFDM allows for high spectral efficiency by effectively utilizing the available bandwidth. With multiple sub-carriers operating in parallel, OFDM can transmit multiple data streams simultaneously, increasing the data rate and overall network capacity [14,15]. This is particularly beneficial for WSNs that require real-time transmission of large amounts of data, such as in smart irrigation systems where sensor nodes need to transmit sensor measurements and control commands [1].

Moreover, OFDM provides resilience against frequency-selective fading, a common challenge in wireless communication. The frequency diversity inherent in OFDM allows the system to combat fading by distributing the data across multiple sub-carriers [16]. This helps maintain reliable communication even in harsh and dynamic wireless environments, contributing to the robustness and reliability of WSNs [2]. Energy efficiency is a critical consideration in WSNs due to the limited power resources available to sensor nodes. OFDM-based WSNs offer energy-saving benefits by enabling efficient power allocation. The ability to allocate power selectively on each sub-carrier allows for adaptive power control, where power can be allocated according to the channel conditions and the specific communication requirements. This adaptive power allocation optimizes energy consumption, ensuring that power is allocated where it is most needed and reducing unnecessary energy expenditure, ultimately prolonging the sensor nodes’ battery life [17,18]. In summary, the adoption of OFDM in WSNs holds significant importance in improving network performance and achieving energy-efficient operations. By leveraging the benefits of frequency diversity, spectral efficiency, and adaptive power allocation, OFDM-based WSNs can enhance data transmission rates, system reliability, and energy efficiency, making them an attractive choice for various applications, including smart irrigation systems and other resource-constrained WSN deployments.

In this context, this paper presents hybrid power allocation algorithms for WSN-based smart irrigation systems using OFDM. The proposed algorithms dynamically adjust the modulation type and power allocation on each sub-carrier, taking advantage of the frequency selectivity of the channel. This adaptive power allocation approach aims to optimize system performance while ensuring energy efficiency and prolonging the sensor nodes’ lifetime. Furthermore, this paper considers practical cases of interest, such as Rate Maximization (RM) to cater to various application requirements. The Greedy Power Allocation (GPA) algorithm, known for its rate-optimal properties, is introduced along with Uniform power allocation (UPA) algorithm, providing options for efficient implementations in resource-constrained WSNs [10,19].

Additionally, the paper addresses the challenges posed by fast fading channels, which can adversely affect the performance of adaptive power allocation algorithms. To mitigate these effects, a linear frequency domain Maximum Ratio Combining pre-coding (Pre-MRC) technique is proposed. This technique maximizes the received sub-carrier Signal-to-Noise Ratio (SNR) and simplifies receiver complexity, enhancing the performance of the proposed power allocation algorithms in hostile environments. Through extensive simulations and analysis, the effectiveness of the proposed algorithms is evaluated in terms of power consumption, system performance, and the extension of the sensor nodes’ lifetime. The results demonstrate the significant benefits of adaptive power allocation in achieving energy efficiency and enhancing the performance of WSN-based smart irrigation systems. Overall, this paper aims to contribute to the development of hybrid energy-efficient algorithms that enhance the lifetime of sensor nodes, thereby improving the sustainability and efficiency of smart irrigation systems based on WSNs. In summary, this work introduces the following contributions:

1. Proposal of a linear frequency domain Maximum Ratio Combining pre-coding (Pre-MRC) technique to mitigate the effects of fast fading channels. This technique maximizes the received sub-carrier SNR and simplifies receiver complexity, improving the performance of the proposed power allocation algorithms in hostile environments.
2. Proposing hybrid power allocation algorithms for WSN-based smart irrigation systems using OFDM. The proposed hybrid algorithms integrate pre-coding with low-complexity power allocation techniques such as Greedy power allocation (GPA) and Uniform power allocation (UPA) techniques. These algorithms dynamically adapt the modulation type and power allocation on each sub-carrier, leveraging the frequency selectivity of the channel to enhance system performance and energy efficiency.
3. Evaluation of the proposed algorithms through extensive simulations and analysis, demonstrating their effectiveness in terms of throughput, energy efficiency, and the extension of the sensor nodes’ lifetime.
4. The significance of achieving energy efficient WSNs in enhancing the sustainability and efficiency of smart irrigation systems.

Through these contributions, this paper aims to advance the field of WSN-based smart irrigation systems, providing efficient solutions for achieving energy efficiency and optimizing the performance of sensor networks in agricultural applications.

## 2. Related work

Several studies have addressed the power allocation challenges in WSNs and their application in smart irrigation systems. In this section, we provide an overview of the relevant works, discussing their methodologies, advantages, disadvantages, and how they compare to the proposed algorithm.

One notable study in [20] proposed a static power allocation scheme for WSN-based smart irrigation systems. The algorithm allocated equal power to all sub-carriers, regardless of the channel conditions. While this approach is straightforward to implement, it fails to exploit the frequency selectivity of the channel, resulting in suboptimal system performance in terms of throughput. Another approach, presented in [21] introduced a dynamic power allocation algorithm based on channel quality estimation in OFDM-based WSNs. The algorithm adjusted the power allocation based on the measured SNR values across the sub-carriers. Although this adaptive approach improved system performance, it required accurate SNR estimation and feedback, which could introduce complexity and overhead.

Chen et al. [22] proposed the Rate-Optimal Power Allocation (ROPA) algorithm for OFDM-based WSNs. This algorithm aimed to maximize the achievable data rate while satisfying a total power constraint. ROPA utilized an iterative optimization process to allocate power across the sub-carriers. However, the computational complexity of ROPA increased significantly with the number of sub-carriers, limiting its scalability. In a different study, the authors in [23] introduced the Grouped Power Allocation (GPA) algorithm, which performed power allocation in groups of sub-carriers. This sub-optimal approach reduced computational complexity compared to the optimal ROPA algorithm while achieving reasonable performance gains. However, GPA did not consider dynamic adaptation of the modulation type on each sub-carrier.

Compared to the aforementioned works, the proposed power allocation algorithm in this study offers several advantages. It leverages the frequency selectivity of the channel to dynamically adapt the modulation type and power allocation on each sub-carrier, resulting in significant performance gains compared to static schemes. The algorithm considers practical cases of RM, providing flexibility to meet different application requirements. Furthermore, it introduces the GPA algorithm, striking a balance between performance and complexity. The inclusion of the linear frequency domain Pre-MRC technique further enhances the algorithm’s performance in fast fading channels.

In addition to the discussed works, there exist other studies in the field of power allocation for WSN-based smart irrigation systems. For example, Li et al. [24] proposed an energy-aware power allocation algorithm that takes into account the energy constraints of the sensor nodes. Their algorithm aimed to maximize the network lifetime by optimizing power allocation and scheduling transmission activities. Although effective in energy conservation, this approach did not consider the modulation type adaptation and frequency selectivity of the channel. In summary, the proposed algorithms outperform the related works by providing adaptive power allocation, considering modulation type adaptation, and achieving a balance between performance and complexity. It addresses the limitations of static schemes, accurate channel estimation requirements, and high computational complexity. By leveraging the frequency selectivity of the channel and incorporating the Pre-MRC technique, the proposed algorithms offer improved system performance and energy efficiency in the context of WSN-based smart irrigation systems.

In recent years, there have been advancements in power allocation algorithms for WSN-based smart irrigation systems. One notable recent work by Wang et al. proposed a Machine Learning-based Power Allocation (MLPA) algorithm [25]. The MLPA algorithm utilized machine learning techniques to learn the optimal power allocation strategy based on historical data and channel conditions. This approach offered the advantage of adaptability to changing environments and improved performance compared to traditional approaches. However, the MLPA algorithm required a large amount of training data and computational resources for the machine learning model, which could be a challenge in resource constrained WSNs. Table 1 presents a summary of the related work in term of contribution and limitations.

**Table 1:** Summary of related work.

Reference	Key Contribution	Limitations	Comparison to Proposed Work
[20]	Introduced a static power allocation scheme for WSN-based smart irrigation systems.	Fails to exploit channel frequency selectivity, leading to suboptimal throughput.	Proposed algorithms dynamically adapt power and modulation to channel conditions, achieving higher throughput and efficiency.
[21]	Proposed a dynamic power allocation algorithm based on SNR estimation for OFDM-based WSNs.	Requires accurate SNR estimation and feedback, introducing complexity and overhead.	Proposed algorithms achieve similar adaptability without requiring extensive feedback mechanisms.
[22]	Developed the Rate-Optimal Power Allocation (ROPA) algorithm to maximize data rate under power constraints.	High computational complexity limits scalability for large networks.	Proposed pre-GPA algorithm balances performance and complexity, making it more scalable for real-world applications.
[23]	Introduced the Grouped Power Allocation (GPA) algorithm, reducing complexity by grouping sub-carriers.	Does not dynamically adapt modulation for each sub-carrier, resulting in performance limitations.	Proposed algorithms dynamically adapt both power and modulation, improving throughput and energy efficiency.
[24]	Presented an energy-aware power allocation algorithm for maximizing network lifetime.	Did not consider modulation type adaptation or channel frequency selectivity.	Proposed algorithms integrate these aspects, offering better system performance and resource utilization.
[25]	Proposed a Machine Learning-based Power Allocation (MLPA) algorithm for adaptability in changing conditions.	Requires extensive training data and computational resources, challenging for resource-constrained WSNs.	Proposed algorithms achieve adaptability without relying on machine learning, reducing complexity and resource requirements.

Compared to these recent works, the proposed algorithm presents a different approach by leveraging the frequency selectivity of the channel and incorporating modulation type adaptation. By dynamically adapting the power allocation based on the channel conditions, the proposed algorithms can achieve enhanced system performance without the need for extensive training data or complex machine learning models. Furthermore, the proposed algorithms consider practical constraints, such as rate maximization, providing flexibility to meet specific application requirements.

## 3. System model and problem formulation

### 3.1. WSN system model

**Fig 1:**
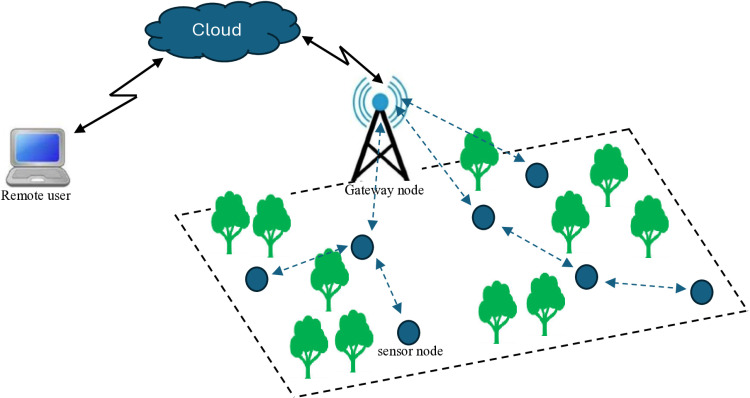
Smart irrigation based WSN system model.

In smart irrigation systems, WSNs play a pivotal role in monitoring and managing agricultural environments. The WSN network model presented in Fig 1 is designed to collect real-time data from various sensor nodes deployed across the agriculture field, which monitor key parameters like soil moisture, temperature, humidity, and light intensity. These nodes communicate wirelessly through a network of sensor nodes, which then relay the data to a central gateway or control unit.

The data is processed to optimize irrigation schedules, ensuring water is used efficiently, reducing waste, and promoting sustainable farming practices. This model enables automated decision-making based on environmental conditions, reducing the need for manual intervention and improving crop yield while conserving resources. Furthermore, the network’s scalability and energy-efficient design are essential for large agricultural fields, making WSNs a core component of modern precision agriculture.

The modelling framework for this study integrates an OFDM-based WSN architecture with adaptive power allocation and pre-coding techniques to optimize system performance. The framework begins with the assumption that sensor nodes operate in a resource-constrained environment, where energy efficiency and network lifetime are critical. The system model, depicted in Fig 1, incorporates key parameters such as sub-carrier SNR, total transmit power *P*_*t*_, and target Bit Error Rate (BER). The proposed framework considers real-world constraints, such as the maximum allowable number of bits per sub-carrier and the energy limitations of sensor nodes.

### 3.2. Problem formulation

**Fig 2:**
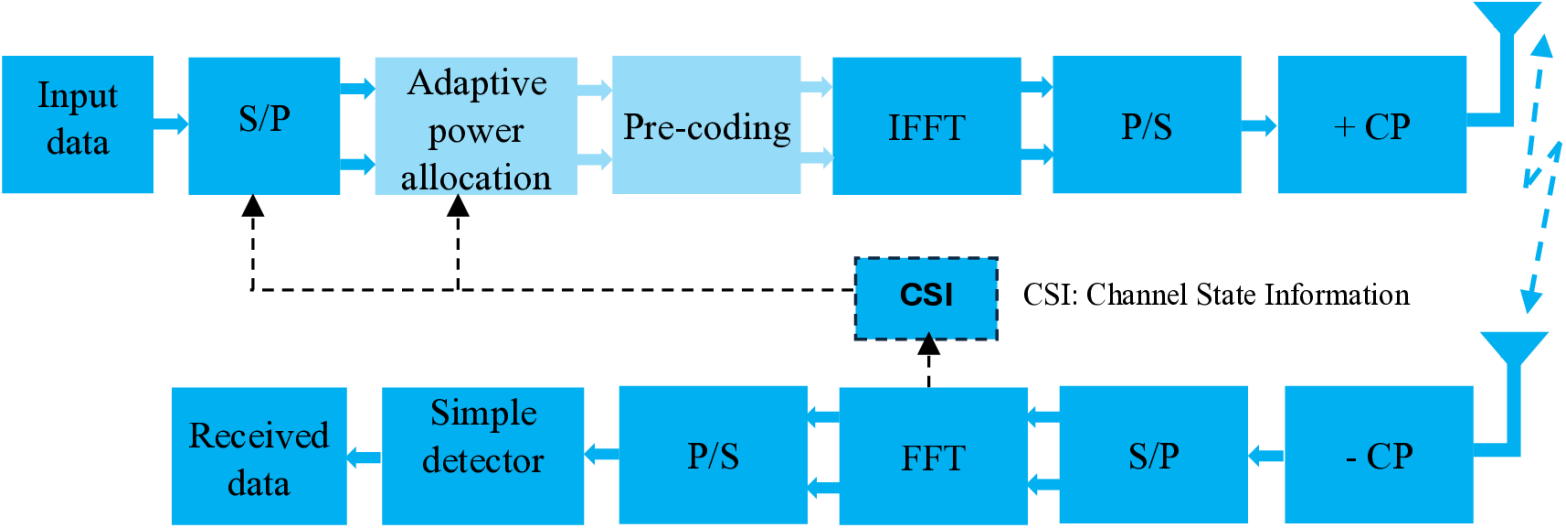
Adaptive pre-coded OFDM-based WSNs architecture.


OFDM has emerged as a promising technique for enabling energy efficient transmission, particularly for WSN-based smart irrigation systems. To enhance the OFDM-based WSNs performance, the utilization of adaptive power allocation algorithms along with frequency domain pre-coding techniques has gained significant attention. In this paper, we propose a hybrid architecture that combines adaptive pre-coded and power allocation techniques for OFDM-based WSNs, as illustrated in Fig 2. The system begins by feeding the data bit stream into a serial-to-parallel (S/P) block, preparing it for subsequent stages of adaptive bit and power allocation. This allocation process adaptively assigns the total transmit power and bits to the available sub-carriers. The adaptive bit and power allocation algorithm efficiently maps the allocated bits *R*_*n*_ on the *nth* sub-carrier to the corresponding QAM symbol (Quadrature Amplitude Modulation), accompanied by its designated transmit power *p*_*n*_. To compensate for channel effects and further enhance system performance, pre-coding is then employed. The received signal undergoes cyclic prefix (CP) removal and Fast Fourier Transform (FFT), resulting in its representation as follows:

Y=HGX+Z (1)

where X is an N×1 column vector of the frequency domain transmitted signal, G is an N×N diagonal matrix of the linear frequency domain pre-coding coefficients, H is an N×N diagonal matrix of the frequency-domain channel coefficients, and Z is the N×1 vector of zero-mean AWGN with variance $\sigma_{z}^{2}$ and *N* represents the number of sub-carriers. Through this proposed architecture, we aim to optimize power allocation and improve the overall performance of the OFDM based-WSNs, particularly in the context of smart irrigation application based on WSNs [Fig 2].

The *nth* sub-carrier undergoing the gain $g_{n}$ that used to send $R_{n}$ bits per symbol. The rate maximization (RM) optimization problem of the OFDM-based WSN can be formulated as

Objective


$$\max_{p_{n}}\;\sum_{n=1}^{N}R_{n}$$ (2)

Constraints


$$\begin{array}{cc} C1:\; & \sum_{n=1}^{N}p_{n}\leq P_{t},\; \end{array}$$

$$\begin{array}{cc} C2:\; & p_{n}\geq 0,\;\forall n\;:\;1\leq n\;\leq N \end{array}$$ (3)

$$\begin{array}{cc} C3:\; & {𝒫}_{e,n\;}\leq \;{𝒫}_{e,\text{target}\;},\;\forall n\;:\;1\leq n\;\leq N \end{array}$$

$$\begin{array}{cc} C4:\; & R_{n}\leq \;R_{\text{max}},\;\forall n\;:\;1\leq n\;\leq N \end{array}$$

In Equation 3, $R_{n}$ represents the rate and $p_{n}$ the power assigned to the *nth* sub-carrier to obtain a target BER of ${𝒫}_{e,n}$, $P_{t}$ represents the total transmit power, ${𝒫}_{e,\text{target }}$denotes the target BER, and $R_{\text{max}}$ represents the maximum allowable no. of bits assigned per sub-carrier.

The carrier-to-noise ratio (CNR) of the *nth* sub-carrier is defined as follows:


$${CNR}_{n}=\frac{g_{n}^{2}}{\sigma_{z}^{2}}$$ (4)

where $\sigma_{z}^{2}$ represents the receiver noise power. The *nth* sub-carrier SNR is given as:


$$\gamma_{n}=p_{n}\times \;{CNR}_{n}$$ (5)

The minimum SNR required to allocate symbols with $R_{l}=\log_{2}M_{l}\;$bits to the *nth* sub-carrier is given as [10]:


$$\gamma_{l}^{\text{QAM}}=\left\{ \begin{array}{c} \frac{{\left[Q^{-1}\left(P_{e,\text{target}}\right)\right]}^{2}}{2}\text{ for BPSK }\backslash \;\backslash \frac{M_{l}-1}{3}{\left[Q^{-1}\left(\frac{1-\sqrt{1-\log_{2}M_{l}\cdot P_{e,\text{target}}}}{2(\;1-\frac{1}{\sqrt{M_{l}}})}\right)\right]}^{2}\text{ for M-QAM} \end{array}\right.\;$$ (6)

where $M_{l}\;$represents the used QAM constellation and $Q^{-1}\;$is the inverse of Q function. The optimization problem of the above constrained will be considered next.

### 4. Pre-coding and power allocation for OFDM-based WSNs

Pre-coding and power allocation are widely utilized signal pre-processing techniques that contribute to enhance OFDM-based WSN systems. Pre-coding addresses the fading issues inherent in wireless channels by improving the received SNR of transmitted signals, thus bolstering system performance. Conversely, power allocation is a signal pre-processing technique that leverages the diversity offered by sub-channels in OFDM-based WSN systems. It dynamically transmits signals through these sub-channels based on their measured SNRs, optimizing the overall transmission efficiency. In the next sub-sections, we present a comprehensive explanation of these two signal pre-processing techniques and their respective roles in improving OFDM-based WSN systems.

### 4.1. Pre-coding techniques

In the upcoming sub-sections, we introduce a variety of receiver-side pre-coding techniques as potential signal pre-processing solutions. These techniques are proposed to effectively pre-code the signals transmitted through OFDM sub-channels, aiming to enhance the overall system performance [26,27].

A Pre-MRC: Maximum ratio combining pre-coding.

The Maximum Ratio Combining Pre-coding (Pre-MRC) technique possesses an attractive characteristic of maximizing SNR at the system receiver. The Pre-MRC involves a diagonal matrix of size N×N, with weighting factors, which can be expressed as [10,27]:


$$G_{\text{pre-MRC}}=H^{H}$$ (7)

where H is an N×N diagonal matrix of the frequency-domain coefficients of the fading channel and $(\cdot {)}^{H}$ is the conjugate transpose of the matrix. Pre-MRC relies on the adjustment of phase and amplitude weighting for individual sub-carriers.

B Pre-EGC: Equal gain combining pre-coding.

The Equal Gain Combining Pre-coding (Pre-EGC) technique focuses on phase correction for each sub-carrier without modifying the amplitude. The Pre-EGC utilizes an *N*×*N* diagonal weighting matrix, which can be represented as [10,28].


$$G_{\text{pre-EGC}}=\frac{H^{H}}{\left|H\right|}$$ (8)

C Pre-ZF: Zero-forcing pre-coding.

Zero-Forcing Pre-coding (Pre-ZF), also known as Orthogonality Restoring Combining (ORC) or channel inversion (CI), focuses on both amplitude and phase correction of the frequency response of the channel. The Pre-ZF utilizes an *N*×*N* diagonal weighting matrix, which can be represented as [27].


$$G_{\text{pre-ZF}}=\frac{1}{H}=\frac{H^{H}}{{\left|H\right|}^{2}}$$ (9)

D Pre-MMSE: Minimum mean square error pre-coding.

Pre-MMSE operates by reducing the Mean Square Error (MSE) among the received and transmitted information, thereby mitigating Inter-Symbol Interference (ISI). The Pre-MMSE employs an *N*×*N* diagonal weighting matrix, which can be written as [29].


$$G_{MMSE}=\frac{H^{H}}{\left({\left|H\right|}^{2}+\frac{1}{SNR}\right)}$$ (10)

### 4.2. The proposed hybrid pre-coding and power allocation algorithms

While power allocation techniques used in OFDM-based WSNs have been extensively investigated in the literature, the integration of power allocation techniques with pre-coding remains a relatively recent area with numerous open challenges. This sub-section aims to address this gap by proposing and examining hybrid algorithms that integrate pre-coding with low-complexity power allocation techniques such as Greedy power allocation (GPA) and Uniform power allocation (UPA) techniques [10]. It is evident that linear pre-coding serves as an effective and low complexity signal pre-processing method for combating channel fading and improving system performance. In this study, the Pre-MRC pre-coding technique is selected to be used in conjunction with the power allocation algorithms (GPA and UPA) due to its ability to leverage the diversity of channel paths, resulting in improved channel SNR and enhanced power consumption which improve WSN lifetime.

By substituting the Pre-MRC pre-coding matrix, as given in Equation 7, into Equation 1, the pre-coded signal at receiver is expressed as follows:


$$Y=HH^{H}X+Z$$ (11)

where $H_{eff}=HG_{\text{pre-MRC}}=HH^{H}$ is the pre coded OFDM effective frequency domain channel. The *nth* sub-carrier CNR defined in Equation 4 is reformulated as


$${CNR}_{n,pre}=\frac{{\left|H_{eff,n}\right|}^{2}}{\sigma_{z}^{2}}$$ (12)

In the next sub-sections, efficient and low-complexity pre-coded power allocation algorithms are presented.

#### 4.2.1. Optimal pre-coded GPA (Pre-GPA) algorithm.

The optimal Pre-GPA algorithm enhances the conventional GPA algorithm by incorporating the Pre-MRC technique at the OFDM transmitter. This modification aims to improve the SNRs. The optimal Pre-GPA algorithm begins with the Pre-coded UPA (Pre-UPA) algorithm and utilizes the surplus power from the Pre-UPA algorithm to optimize the power allocation of the system. This optimization process aims to maximize the overall system performance and improve energy efficiency. The subsequent sub-sections provide a comprehensive description of the Pre-GPA algorithms, highlighting their intricacies and functionalities.

A Pre-UPA Algorithm

The Pre–UPA algorithm steps is listed as:

1) Calculate $\gamma_{l}^{\text{QAM}}$ for all $M_{l},\;1\leq l\leq L$, with target BER ${𝒫}_{e,n}={𝒫}_{e,\text{target}}$ using Equation 6.
2) The total power budget $P_{\text{t}}$ is then allocate between all sub-carriers, equally, as shown in Equation 13,

$$\gamma_{n,pre}=p_{n}\times {CNR}_{n,pre}=\frac{P_{\text{t}}}{N}\times \frac{{\left|H_{eff,n}\right|}^{2}}{\sigma_{z}^{2}}$$ (13)

3) Reallocate sub-carriers based on their SNR values $\gamma_{n,pre}$ into QAM groups bounded by QAM levels $\gamma_{l}^{\text{QAM}}$ $\gamma_{k}^{QAM}\;$and $\gamma_{l+1}^{\text{QAM}}\gamma_{k+1}^{QAM}$, i.e.,

$$\gamma_{l}^{\text{QAM}}\leq \gamma_{n,pre}<\;\gamma_{l+1}^{\text{QAM}}$$ (14)

4) Assign QAM constellation $M_{l}$ to the sub-carriers in each $G_{l}\;$group, ensuring that the total number of assigned bits for the group is

$$R_{l}^{pre-UPA}=\sum_{n\in G_{l}}R_{n}$$ (15)

with $R_{0}^{pre-UPA}=0$ $B_{0}^{upa}=0\;B_{0}^{upa}=0$and the total excess power for this group is given as:


$$P_{l,excess}^{pre-UPA}=\sum_{n\in G_{l}}\frac{\left(\gamma_{n,pre}-\gamma_{l}^{\text{QAM}}\right)}{{CNR}_{n,pre}}$$ (16)

5) The total allocated bits of the system and the power consumed by the Pre-UPA algorithm are:

$$R^{pre-UPA}=\sum_{l=1}^{L}R_{l}^{pre-UPA}$$ (17)

$$P_{\text{used}}^{\text{Pre-UPA}}=P_{\text{t}}-P_{excess}^{pre-UPA}=P_{\text{t}}-\sum_{l=1}^{L}P_{l,ex}^{pre-UPA}$$ (18)

The excess power generated by the Pre-UPA algorithm is effectively utilized by several algorithms, serving as a valuable indicator of the efficient use of the total transmit power $P_{\text{t}}$.

The Pre-GPA algorithm builds on the initialization step outlined earlier. It iteratively redistributes the excess power from the Pre-UPA algorithm. Following the procedure in Table 2, the total allocated bits and consumed power are determined as follows:

**Table 2:** The Pre-GPA algorithm operation.

Step	Operation
**Input**	$R_{n}^{\text{Pre-UPA}}\;,\;P_{\text{excess}}^{\text{Pre-UPA}}\;,\;\gamma_{n,pre}$
**Initialization**	Set excess power of Pre-GPA, $P_{excess}^{\text{Pre-GPA}}=P_{\text{excess}}^{\text{Pre-UPA}}$ as in Equation 18. for each sub-carrier do the following: Set $R_{n}^{\text{Pre-GPA}}{=R}_{n}^{\text{Pre-UPA}}$ Initiate $l_{n}=l$ as in (3.24) Calculate the min. required upgrade power: $p_{n}^{up}=\frac{\left(\gamma_{l_{n}+1}^{\text{QAM}}-\gamma_{l_{n}}^{\text{QAM}}\right)}{\;{CNR}_{n,pre}}$
**Iteration**	while $P_{d}^{\text{Pre-GPA}}\geq \text{min}(p_{n}^{up}\backslash text\mathrm{and}\text{min}\left(l_{n}\right)<L$ $j=\text{argmin}\left(p_{n}^{up}\right),\;1\leq n\leq N$ $l_{j}=l_{j}+1,\;P_{excess}^{\text{Pre-GPA}}=P_{excess}^{\text{Pre-GPA}}-p_{j}^{up}$ $\text{If }l_{j}=1$ $R_{j}^{\text{Pre-GPA}}=\log_{2}M_{1},p_{j}^{up}=\frac{\left(\gamma_{2}^{\text{QAM}}-\gamma_{1}^{\text{QAM}}\right)}{\;{CNR}_{j,pre}}$ $R_{j}^{\text{Pre-GPA}}=R_{j}^{\text{Pre-GPA}}+\log_{2}\left(\frac{M_{l_{j}}}{M_{l_{j}-1}}\right),p_{j}^{up}=\frac{\left(\gamma_{l_{j}+1}^{\text{QAM}}-\gamma_{l_{j}}^{\text{QAM}}\right)}{\;{CNR}_{j,pre}}$ else $R_{j}^{\text{Pre-GPA}}=R_{j}^{\text{Pre-GPA}}+\log_{2}\left[\frac{M_{l_{j}}}{M_{l_{j}-1}}\right],p_{j}^{up}=+\infty$ end end $R^{\text{Pre-GPA}}=\sum_{n=1}^{N}R_{n}^{\text{Pre-GPA}}$
**Output**	$R^{\text{Pre-GPA}},\;P_{excess}^{\text{pre-GPA}}$


$$R^{\text{pre-GPA}}=\sum_{n=1}^{N}R_{n}^{\text{pre-GPA}}$$ (19)

and


$$P_{\text{used}}^{\text{pre-GPA}}=P_{\text{t}}-P_{d}^{\text{pre-GPA}}$$ (20)

## 5. Simulation results and discussions

This section presents computer simulations using MATLAB program to assess the performance of the proposed algorithms. The non-pre-coded power allocation algorithms are also included as a benchmark for comparison. The proposed modelling framework was applied to evaluate the performance of the hybrid algorithms through simulations. The simulations consider a network area of 100 × 100 m^2^, with sensor node counts varying from 200 to 1000. The channel is modeled using a 6-tap Finite Impulse Response (FIR) filter, and perfect Channel State Information (CSI) is assumed for accurate SNR calculations. The framework incorporates realistic constraints, such as a fixed power budget and target BER of 10^-3^, to simulate real-world deployment scenarios effectively. To evaluate the performance of the proposed algorithms, the following metrics are considered: throughput, energy efficiency, and network lifetime. Table 3 summarizes the parameters used in simulation.

**Table 3:** Parameters used in simulation and their values.

Parameter	Value
Network area	100 × 100 m^2^
Number of sensors	200:1000
Transmitted energy	50 nJ per bit
Multipath channel (*L*_p_)	6 taps FIR
Sub-carriers no. (*N*)	64
QAM level constellation	[4 16 64 256]
Pre-coding	Pre-MRC
Power allocation algorithms	UPA, GPA
Channel estimation	Perfect CSI

### 5.1. Performance analysis

**Fig 3:**
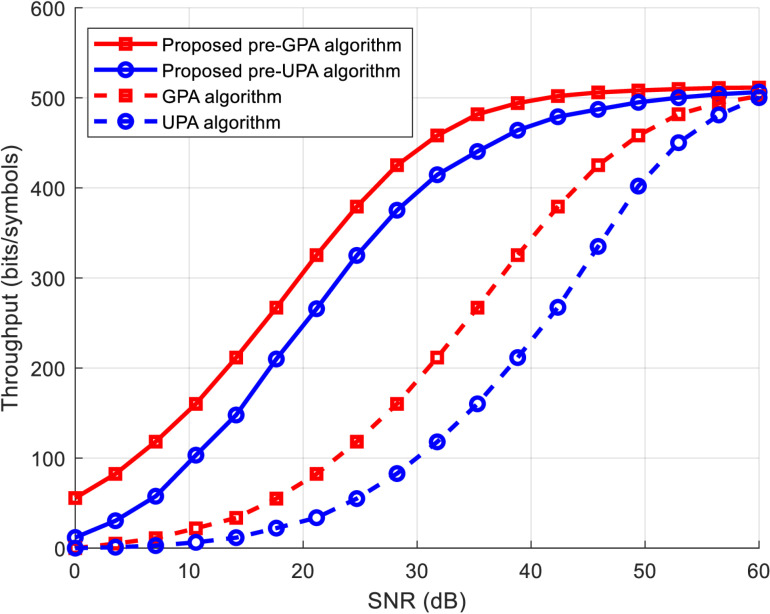
Throughput versus SNR for proposed and conventional power allocation algorithms.

Fig 3 illustrates the achievable throughput as a function of SNR for both the conventional GPA and UPA algorithms, as well as the proposed hybrid pre-GPA and pre-UPA algorithms, with a target BER of 10^-^³ and a 6-tap FIR (Finite Impulse Response) channel. The figure clearly shows that the proposed hybrid algorithms, particularly pre-GPA, outperform the conventional ones. For instance, to achieve a throughput of 400 bits per symbol, the pre-GPA algorithm requires 18 dB less SNR compared to the conventional non-pre-coded GPA algorithm. This SNR reduction leads to lower power consumption for sensor nodes, thereby extending their operational lifetime.

**Fig 4:**
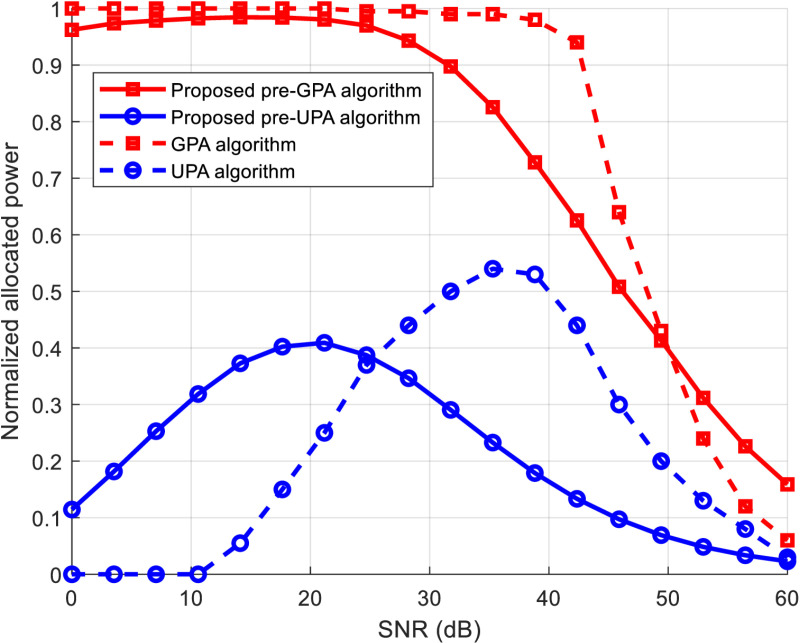
Normalized allocated power against SNR for proposed and conventional power allocation algorithms.

Fig 4 illustrates the normalized allocated power (total allocated power relative to total transmit power) versus SNR for both conventional and proposed hybrid algorithms, with a target BER of 10^-3^ and a 6-tap FIR channel. At low SNR levels, the conventional UPA and the proposed pre-UPA algorithms excel in conserving transmit power, though at the expense of reduced throughput, as shown in Fig 3. GPA-based algorithms, on the other hand, are more effective than UPA algorithms in utilizing transmit power to enhance throughput. At higher SNR levels, the performance of GPA-based algorithms, with and without pre-coding, converges with that of the UPA algorithm due to the increased excess power at the highest QAM level.

### 5.2. Comparative analysis

**Fig 5:**
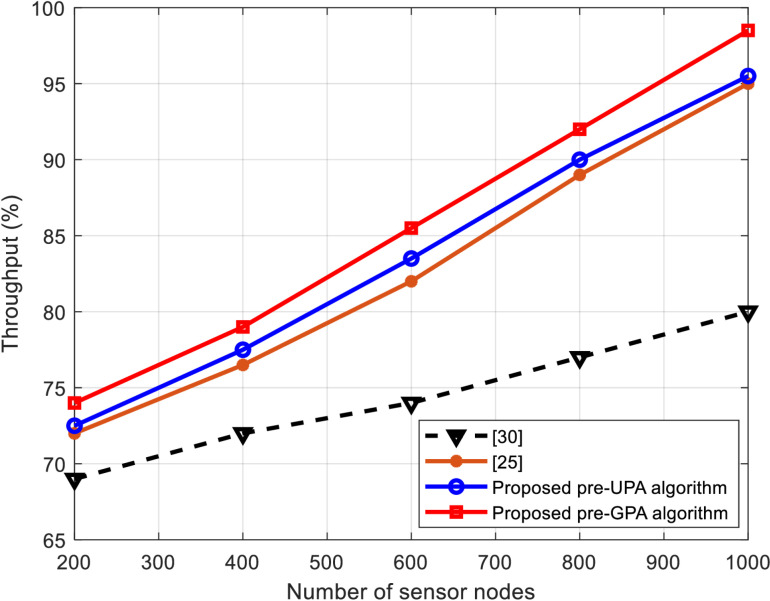
Performance comparison in terms of throughput versus number of sensor nodes.

**Fig 6:**
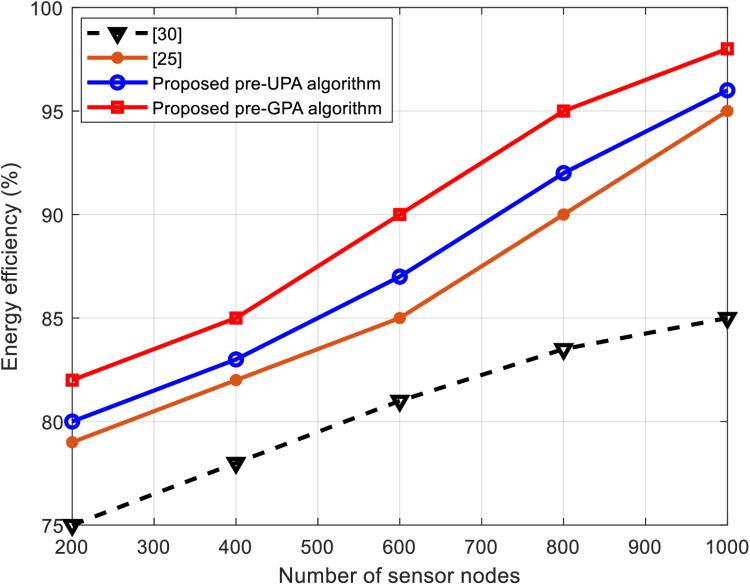
Energy efficiency comparison versus number of sensor nodes.

**Fig 7:**
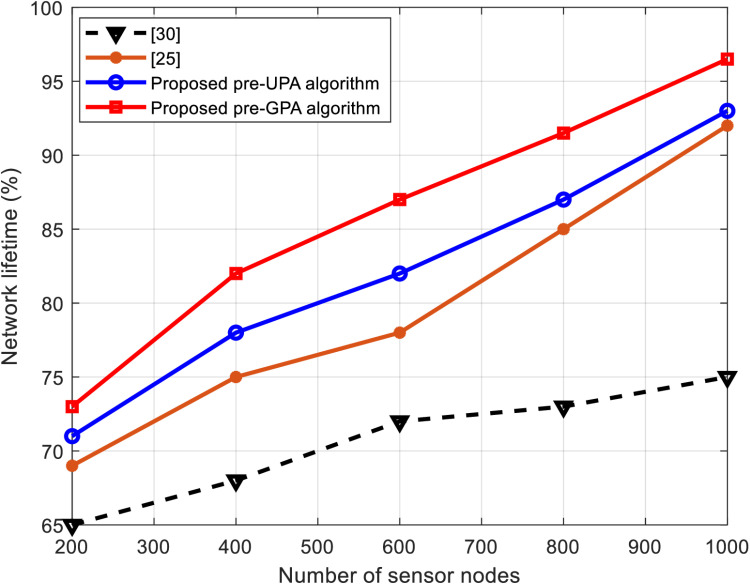
Performance comparison in terms of network lifetime against number of sensor nodes.

This subsection presents a comparative analysis of WSNs using the proposed hybrid algorithms against those from the literature, such as those in [25] and [30]. The comparison is made based on throughput percentage, energy efficiency, and network lifetime, as shown in Figs 5–7, respectively.

Fig 5 highlights the throughput performance as the number of sensor nodes increases. The proposed pre-GPA and pre-UPA algorithms consistently outperform the conventional GPA and UPA approaches. Specifically, the pre-GPA algorithm achieves up to 98.5% throughput, demonstrating its ability to efficiently utilize available channel resources even as the network grows. This superior performance is attributed to the dynamic allocation of power and modulation levels based on the SNR of each sub-carrier, ensuring optimal data transmission across varying conditions. The pre-UPA algorithm, while slightly less effective than pre-GPA, still outperforms conventional methods with a throughput of 96.5%, showcasing its ability to balance simplicity and performance. While the achieved throughput is 95% and 80% for the algorithms in [25] and [30], respectively.

Fig 6 illustrates the energy efficiency of the algorithms as a function of network size. The proposed hybrid algorithms show a marked improvement in energy efficiency compared to conventional approaches. The pre-GPA algorithm demonstrates the highest energy efficiency due to its intelligent redistribution of excess power and effective pre-coding, which minimizes unnecessary energy consumption. For example, with 1000 sensor nodes, the pre-GPA algorithm achieves an energy efficiency of 98%, compared to 95% and 85% for the algorithms presented in [25] and [30], respectively. The results validate the algorithms’ capability to conserve energy while maintaining high performance, making them highly suitable for resource-constrained environments like smart irrigation systems.

Fig 7 examines the network lifetime, a critical metric for the sustainability of WSN-based smart irrigation systems. The proposed pre-GPA algorithm achieves the longest network lifetime, followed closely by the pre-UPA algorithm. This outcome is a direct result of the efficient energy usage enabled by the hybrid algorithms, which allocate power adaptively and optimize resource utilization. Compared to conventional GPA and UPA algorithms [19] and the algorithms presented in [25] and [30], the hybrid methods significantly reduce power wastage and extend the operational lifespan of sensor nodes, particularly in larger networks.

Table 4 presents a comparison of performance metrics for proposed and existing algorithms at 1000 sensor nodes. The proposed algorithms achieve the highest performance due to efficient power allocation and pre-coding techniques.

**Table 4:** Comparison of performance metrics for proposed and existing algorithms.

Algorithm	Throughput (%)	Energy Efficiency (%)	Network Lifetime (%)
Proposed Pre-GPA	98.5	98.0	96.5
Proposed Pre-UPA	96.5	96.0	93.0
Algorithm in [25]	95.0	95.0	92.0
Algorithm in [30]	80.0	85.0	75.0

### 5.3 Comparison of computational complexity

This subsection provides a comparison of the computational complexity of the algorithms discussed in this paper, as summarized in Table 5.

**Table 5:** The computational complexity of each algorithm.

Algorithm	Computational Complexity	Remarks
Proposed Pre-GPA	*O*(*I* ⋅ *N*)	Balances complexity and performance, suitable for real-time applications.
Proposed Pre-UPA	*O*(*N*)	Low-complexity, ideal for scenarios with constrained computational resources.
Conventional GPA	*O*(*I* ⋅ *N*)	Moderate complexity but lacks pre-coding benefits, resulting in lower performance.
Conventional UPA	*O*(*N*)	Simple but static, resulting in suboptimal system performance.
ROPA [22]	*O*(*I* ⋅ *N*^*2*^)	High complexity, limiting scalability for larger networks
MLPA [25]	Training: *O*(*D* ⋅ *M*^*2*^), Inference: *O*(*N*)	High adaptability but requires significant computational and resource investment for training
Algorithm in [30]	*O*(*G* ⋅ *N*), where *G* is the number of groups	Groups sub-carriers to reduce complexity but lacks dynamic modulation adaptation for each sub-carrier.

A **Proposed algorithms.**
  1 **Pre-GPA Algorithm**

- **Pre-GPA Algorithm:** The computational complexity of the Pre-GPA algorithm is primarily determined by:
- **Initialization Step:** The equal allocation of power across sub-carriers, which has a complexity of *O*(*N*), where *N* is the number of sub-carriers.
- **Iterative Redistribution:** This step involves recalculating power for each sub-carrier until convergence, with complexity *O*(*I*⋅*N*), where *I* is the number of iterations.
- **Overall Complexity:** The total complexity can be approximated as *O*(*I*⋅*N*), making it suitable for real-time applications with moderate computational resources.
  1. **Pre-UPA Algorithm:**
- **Equal Power Allocation:** A simpler variant with complexity *O*(*N*), as it avoids iterative redistribution.
- **Overall Complexity:** *O*(*N*), making it a low-complexity option, ideal for scenarios with limited computational capacity.

B **Conventional Algorithms:**
  1. **GPA Algorithm**

- Performs iterative power redistribution without pre-coding, with complexity *O*(*I*⋅*N*).
- Slightly less complex than the Pre-GPA algorithm due to the absence of pre-coding computations but achieves lower performance.
  1. **UPA Algorithm**
- Fixed power allocation with complexity *O*(*N*), similar to the Pre-UPA algorithm but lacks dynamic adaptation.

C **Algorithms from literature:.**
  1. **ROPA Algorithm (from [22])**:

- An iterative optimization algorithm with complexity *O*(*I*⋅*N*^*2*^), due to its reliance on solving optimization problems for each sub-carrier iteratively.
- Offers high performance but at the cost of scalability for large networks.
  1. **MLPA Algorithm (from [25]):**
- Employs machine learning techniques with a complexity dependent on the training phase and inference step. The training phase is computationally intensive (*O*(*D*⋅*M*^*2*^), where *D* is the dataset size and *M* is the model size, while inference has a complexity of *O*(*N*).
- High adaptability but resource-intensive, making it less practical for resource-constrained WSNs.
  1. **The Algorithm in [30]:**
- Groups sub-carriers into *G* groups and applies power allocation to each group, significantly reducing computational complexity compared to individual sub-carrier optimization.
- **Complexity**: *O*(*G*⋅*N*), where *G* is much smaller than *N*, making it more efficient than *O*(*I*⋅*N*^*2*^) algorithms like ROPA. However, this approach sacrifices fine-grained adaptation, leading to slightly reduced performance in scenarios with highly variable channel conditions.

**Fig 8:**
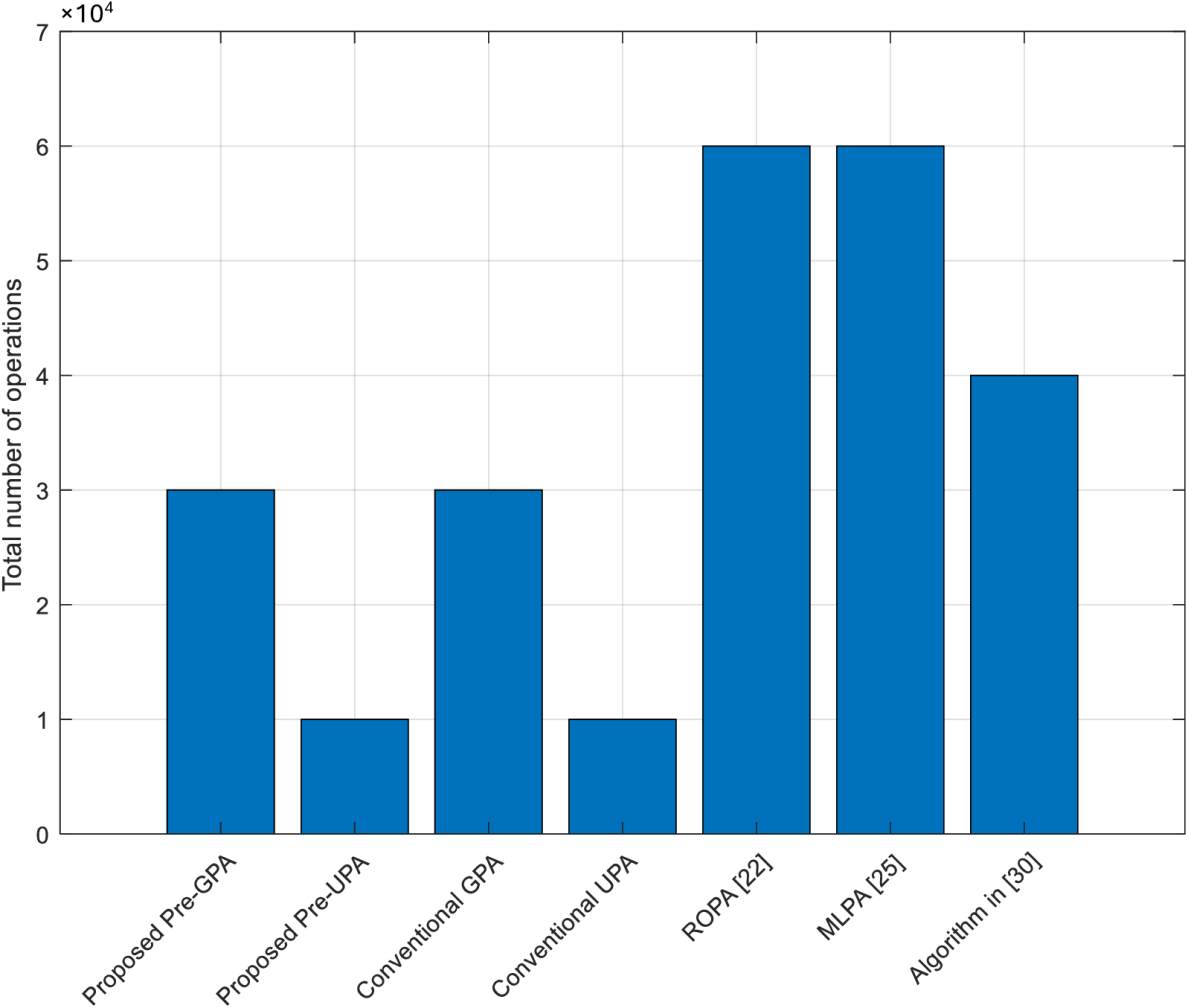
The computational complexity comparison of the considered algorithms.

The computational complexity comparison presented in Fig 8 highlights the trade-offs between the performance and practicality of various algorithms. The proposed pre-GPA algorithm strikes a balance between complexity and performance, making it suitable for real-time applications, while the proposed pre-UPA algorithm offers a low-complexity alternative ideal for resource-constrained environments. In contrast, conventional algorithms such as GPA and UPA lack the benefits of pre-coding and adaptive power allocation, resulting in moderate or suboptimal performance despite manageable complexity levels. The ROPA algorithm [22] delivers high performance but is hindered by its scalability due to significantly higher complexity. Similarly, the MLPA algorithm [25] demonstrates adaptability but requires extensive training and computational resources, making it less practical for real-time or resource-constrained deployments. The algorithm in [30], which groups sub-carriers, reduces computational demands but sacrifices fine-grained modulation adaptation, leading to limited performance improvements. Overall, the proposed algorithms provide an optimal trade-off between computational efficiency and system performance, making them suitable for WSN-based smart irrigation systems.

## 6. Conclusion

This paper presented enhanced hybrid pre-coding and power allocation algorithms for OFDM-based WSNs used in smart irrigation systems. By integrating pre-coding techniques with low-complexity power allocation, the proposed algorithms address the limitations of static schemes and deliver significant performance improvements. The Pre-MRC pre-coding technique was shown to be an effective method to mitigate channel fading, while the dynamic adjustment of modulation type and power allocation across sub-carriers, based on SNR values, led to optimized system performance. Two hybrid pre-coded power allocation algorithms are introduced, pre-GPA and pre-UPA algorithms. The obtained results demonstrated that the hybrid pre-GPA algorithm could reduce the required SNR by up to 18 dB to achieve a target throughput of 400 bits per symbol, outperforming conventional algorithms in terms of throughput, energy efficiency, and network lifetime. The pre-GPA algorithm achieved up to 98.5% throughput while extending system longevity, highlighting its potential for enhancing the sustainability of smart irrigation systems.

As a future work, integrating machine learning techniques into power allocation and modulation adaptation algorithms may further enhance system adaptability and energy efficiency. Research could also be extended to investigate the potential of combining these approaches with renewable energy sources, such as solar-powered sensor nodes, could contribute to even greater sustainability in smart agriculture.

## Supporting information

S1 Data Figure data. (XLSX)
